# A Needle Guidance Device Improves the In-Plane Ultrasound-Guided Imaging in Medical Students' Education

**DOI:** 10.7759/cureus.86209

**Published:** 2025-06-17

**Authors:** Tetsuro Kimura, Masahiko Ohashi, Atsushi Kobayashi, Akira Suzuki, Yoshiki Nakajima, Hiroyuki Kinoshita

**Affiliations:** 1 Department of Anesthesiology and Intensive Care, Hamamatsu University School of Medicine, Hamamatsu, JPN

**Keywords:** medical education, needle guidance device, phantom study, the in-plane technique, ultrasound-guided needle insertion

## Abstract

Background: Clinicians apply the ultrasound-guided technique to securely access blood vessels and peripheral nerves in various medical conditions. Acquiring the in-plane technique for this purpose is challenging for novice medical practitioners.

Objectives: We aimed to assess whether a needle guidance device improves the needle puncture speed and the quality of the needle visualization in the ultrasound-guided in-plane technique on both horizontal and inclined surfaces by medical students, compared with freehand.

Methods: We set the following three ultrasound-guided techniques: the needle direction along the visual axis in the conditions of the horizontal plane of the phantom, the plane inclined to the right at 45 degrees, and the plane inclined to the left at 45 degrees. Right-handed 20 medical students were asked to implement ultrasound-guided punctures with and without a needle guidance device (SIVA guide™, Fuji-Medical Co., Tokyo, Japan) in the three conditions.

Results: Under all puncture settings, the time it took to reach the set success point of the target in the ultrasound-guided punctures with the needle guidance device was less than (up to 4.14 sec (median); *P*<0.01) in the punctures without the device. The needle image quality score and the participants' self-assessment of the procedure difficulty score in the punctures with the needle guidance device were better than those without it.

Conclusion: A needle guidance device appears to improve the needle puncture speed and the quality of the needle visualization in the ultrasound-guided in-plane technique on both horizontal and inclined surfaces by novice medical students, compared with freehand.

## Introduction

Clinicians securely apply ultrasound-guided needle access to blood vessels and peripheral nerves in various medical conditions [1-3]. The accurate and safe needle advancement is indeed essential to the technique, providing the real-time visualization of blood vessels, nerves, and surrounding structures [1-3]. Therefore, understanding ultrasound anatomy and accurately handling the puncture needle with the ultrasound probe is critical for novice medical practitioners to implement the ultrasound-guided technique appropriately.

Ultrasound-guided needle access possesses two techniques, including the in-plane (or parallel) and out-of-plane (or cross-sectional) approaches [4]. Of these techniques, the ultrasound beam should contact the needle at a near-perpendicular angle in the in-plane technique. Also, the needle path must remain in-plane with the ultrasound probe, allowing the continuous visualization of the entire needle shaft and the tip from the insertion site to each target. However, acquiring this technique is demanding for novice medical practitioners since they need to adjust the entire needle with the thin ultrasound beam throughout the procedure [5-7].

Previous studies documented the utility of needle guidance devices, by which an insertion needle is kept in-plane with the ultrasound beam, and thus, the operator could advance the needle through the device in the ultrasound-guided in-plane approach [8-10]. However, the above studies were implemented under limited conditions, where the phantom surface was set horizontally [8-10]. Thus, whether the needle guidance device use helps acquire the ultrasound-guided technique when the operators have to insert the needle via an inclined surface is unclear. Also, whether the needle guidance device adds some advantage to acquiring the ultrasound-guided in-plane technique for novice clinical practitioners is unknown.

Therefore, the current study aimed to assess whether a needle guidance device improves the needle puncture speed and the quality of the needle visualization in the ultrasound-guided in-plane technique on both horizontal and inclined surfaces by novice medical students, compared with freehand.

## Materials and methods

The study was performed from October to December 2016 at the Department of Anesthesiology, Hamamatsu University School of Medicine, Hamamatsu, Japan. Ethical approval for this study (IRB approval number 16-136) was provided by the Hamamatsu University School of Medicine Research Ethics Board, Hamamatsu, Japan, on December 24, 2015. This study was registered in the University Hospital Medical Information Network Clinical Trials Registry (UMIN000024247). Written informed consent was obtained from all medical students before their study enrollment. The procedures in the current study followed the "Declaration of Helsinki" and the ethical standards of the responsible committee on human experimentation. Twenty medical students of Hamamatsu University School of Medicine were recruited for the current study. The students were in their fifth clinical year of education. They had previously received theoretical lessons regarding anesthesiology but had no experience with simulation training for ultrasound-guided vascular access and peripheral nerve block.

**Figure 1:**
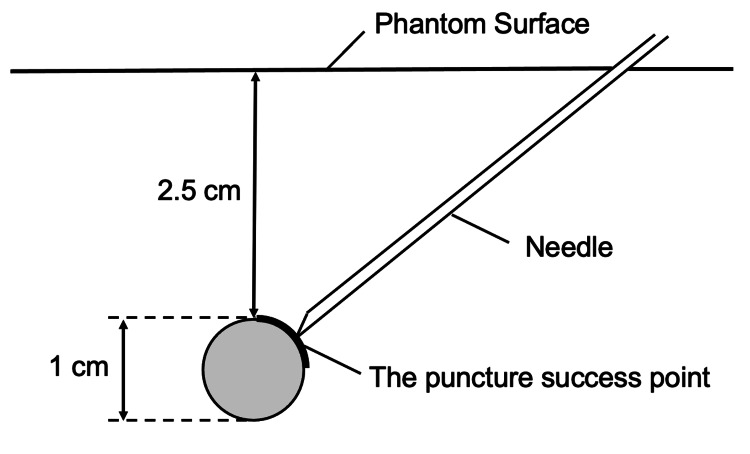
The puncture success point The puncture success point was set at the proximal quarter, 2.5 cm below the phantom surface.

We employed a high-definition ultrasound apparatus equipped with a multifrequency linear probe set at 15 MHz resolution (S-Nerve™, HFL50x/15-6 MHz; Sonosite Inc., Bothell, Washington, United States) to acquire ultrasound images in the present study. An investigator (Masahiko Ohashi) optimized the depth and gain settings of the ultrasound apparatus to obtain appropriate images. We adopted a gel phantom (AGL800™; ALFABIO Inc., Gunma, Japan), a short bevel echogenic 22G needle (PM-Echo™; Hakko Inc., Tokyo, Japan), and a needle guidance device (SIVA guide™; Fuji-Medical Co., Tokyo, Japan) for the following simulated ultrasound-guided puncture techniques. The SIVA guide™ was developed for universal use with various ultrasound probes. It allows the operator to keep a puncture needle in-plane with a thin ultrasound beam without restricting the needle angulation and direction. It consists of a guiding portion and a bracket, both of which are latex-free. The puncture target was an embedded vascular-like structure 1 cm in diameter in the phantom. The puncture success point, at which the needle tip reached, was set at the proximal quarter, 2.5 cm below the phantom surface (Figure 1).

**Figure 2:**
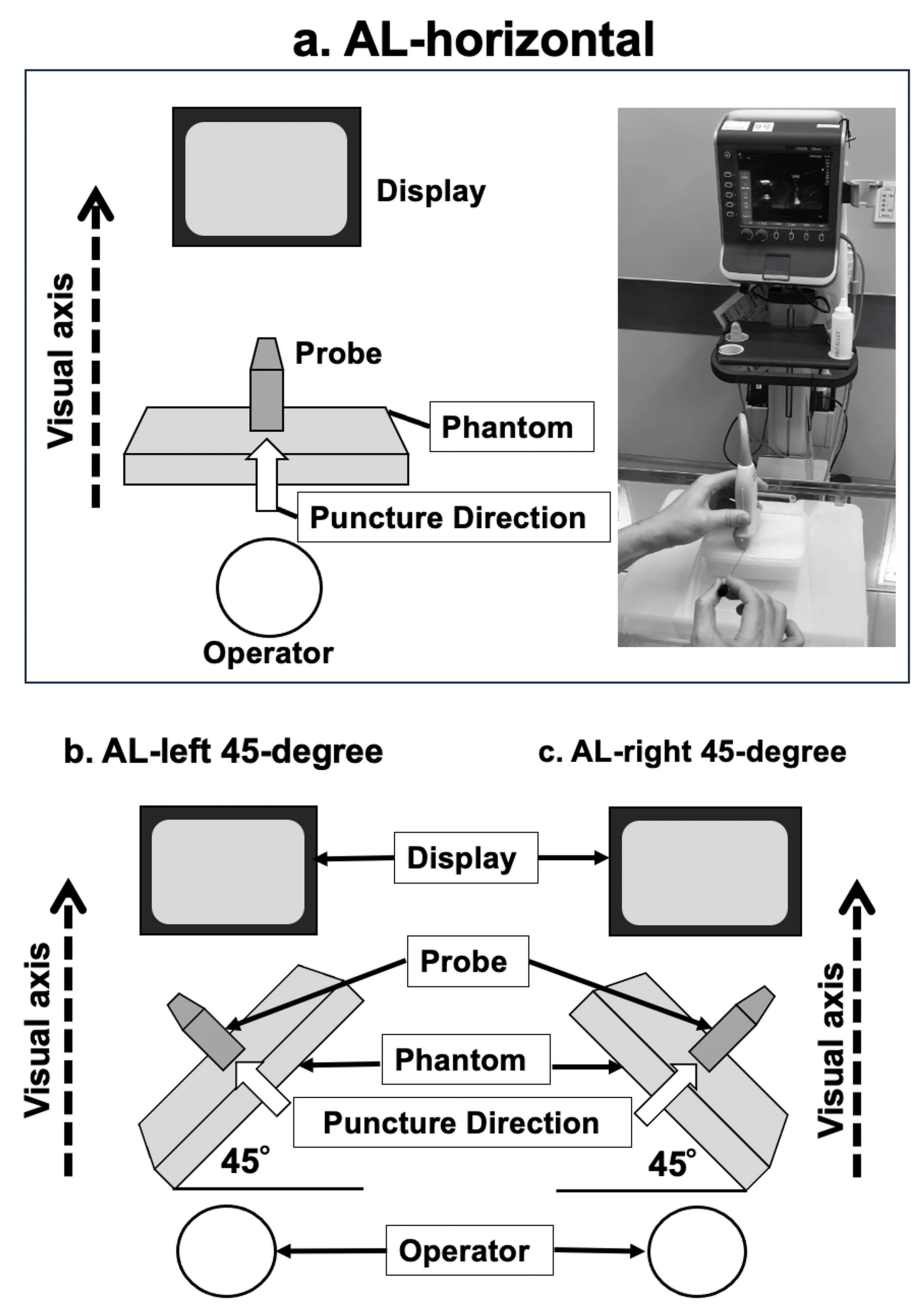
The settings of puncture procedure The actual set-up in the current study, including a gel phantom (Phantom), a multifrequency linear ultrasound probe (Probe), the ultrasound monitor (Monitor), and the participant position (Operator), is shown. Also, the operator's visual axis with puncture direction and the inclined degrees toward the left and right 45 degrees each are indicated (a-c). Each participant performed ultrasound-guided punctures both with and without the SIVA™ guide, under the following three conditions: (a) puncture on a horizontal plane (AL-horizontal group), (b) puncture on a plane inclined 45 degrees to the right (AL-right 45-degree group), and (c) puncture on a plane inclined 45 degrees to the left (AL-left 45-degree group). In all conditions, the needle was inserted along the ultrasound visual axis.

Before the commencement of the study, we asked all medical students to watch a 20-minute educational video about the methodology. The educational video included information regarding the purpose of the current study, the ultrasound principle, how participants advanced the needle used in the present study, and how to use the needle guidance device appropriately. We did not check participants' level of understanding after watching the video. The current study focused on evaluating whether the needle guidance device use helps acquire the ultrasound-guided technique more efficiently when the operators have to insert the needle via an inclined surface. Therefore, we set the following three ultrasound-guided techniques. We defined three ultrasound-guided puncture conditions for this study: (1) puncture along the visual axis on a horizontal plane (AL-horizontal group), (2) puncture on a plane inclined 45 degrees to the right (AL-right 45-degree group), and (3) puncture on a plane inclined 45 degrees to the left (AL-left 45-degree group) (Figure 2). Each participant was asked to implement ultrasound-guided punctures with and without the SIVA guide™ in three conditions three times each before the actual measurements.

The primary study outcome was whether the time reaching the set success point of the target in the ultrasound-guided punctures differed between the conditions with and without the SIVA guide™ among groups in those where the plane was inclined. The secondary outcomes included the number of needle direction changes, the needle image quality using a four-point score (0-3, Table 1) [6], and the participants' self-assessment of the procedure difficulty using a four-point score (0-3: 0=easy, 1=normal, 2=difficult, and 3=extremely difficult). All participants were not informed about the above evaluation criteria.

**Table 1:** Quality of needle imaging

Score		Description
0	Excellent	The needle shaft and tip are fully visualized as it contacts the target
1	Good	The needle tip and part of the shaft are visualized as it contacts the target
2	Unsatisfactory	The needle tip is not visualized as it contacts the target, the tip position is indicated by tissue movement, but all or part of the needle shaft is seen at time of contact with the target
3	Poor	Contact with the target occurs without visualization of the needle tip or the needle is detected (by tissue movement) to have passed deep to the target without needle visualization

Upon the data collection, each participant executed three punctures at two patterns with and without the SIVA guide™. We determined the puncture success as when the ultrasound image evaluator (Tetsuro Kimura) noted that the needle tip reached the success point of the target. Also, the number of needle direction changes, the needle image quality using a four-point score, and the participants' self-assessment of the procedure difficulty using a four-point score were recorded. The ultrasound image evaluator (Tetsuro Kimura), blinded to the puncture settings, assessed the puncture success and quality of needle visibility only from the ultrasound monitor view. The sequence of puncture settings, including assigned groups and patterns with and without the SIVA guide™, was decided in advance using a random number table on the computer, and the information was blinded to the ultrasound image evaluator.

Sample size determination

We used G*Power Version 3.1.9.6 (Heinrich-Heine-Universität Düsseldorf, Düsseldorf, Germany) for the power calculation. We calculated that 15 students per group would need 80% power at a two-sided α level of 0.05, a β error of 0.2, and an effect size d of 0.8 regarding the time difference of four seconds to reach a target between freehand and the needle guidance device use. Accordingly, the enrolment of medical students was considered adequate.

Statistical analysis

Statistical analyses were performed using IBM SPSS Statistics for Windows, Version 27.0 (Released 2019; IBM Corp., Armonk, New York, United States). The data are shown as the median and interquartile range (IQR). The Wilcoxon signed-rank test was performed for group comparisons, respectively. Differences were considered statistically significant when p<0.05.

## Results

**Figure 3:**
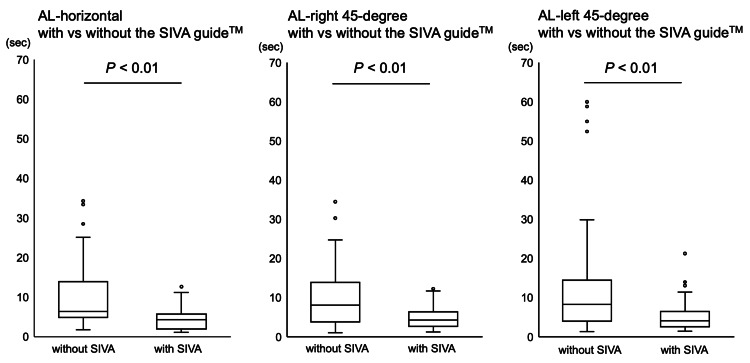
The time reaching the set success point The time reaching the set success point of the target in the ultrasound-guided punctures under the conditions with and without the SIVA guide™ in AL-horizontal, AL-left 45-degree, and AL-right 45-degree groups, respectively, is shown. The data are shown as box-and-whisker plots (maximum and minimum values and IQR) with outliers (white circles).

We recruited 20 (11 male and 9 female) right-handed medical students without exclusion. All punctures were performed within 90 seconds. The time reaching the set success point of the target in the ultrasound-guided punctures with the SIVA guide™ was less compared with the punctures without the SIVA guide™ under all puncture settings (AL-horizontal with 4.33 (1.97-5.72) sec vs. 6.44 (4.86-13.95) sec without the SIVA guide™ (p<0.01); AL-right 45-degree with 4.25 (2.66-6.38) sec vs. 8.11 (3.82-13.88) sec without the SIVA guide™ (p<0.01); AL-left 45-degree with 4.12 (2.56-6.50) sec vs. 8.26 (3.98-14.42) sec without the SIVA guide™ (p<0.01)) (Figure 3).

**Figure 4:**
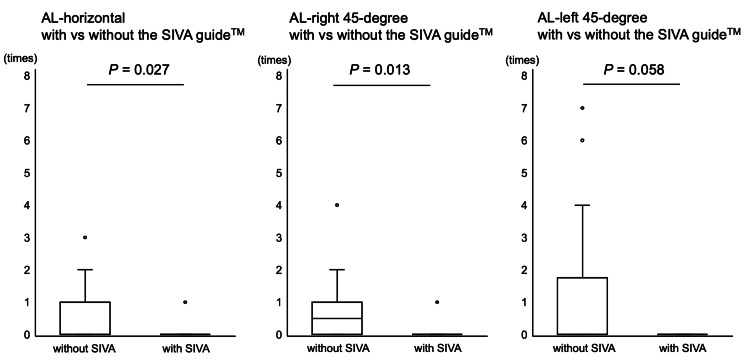
The number of needle direction changes The number of needle direction changes under the conditions with and without the SIVA guide™ in AL-horizontal, AL-left 45-degree, and AL-right 45-degree groups, respectively, is shown. The data are shown as box-and-whisker plots (maximum and minimum values and IQR) with outliers (white circles).

The number of needle direction changes in the punctures with the SIVA guide™ was less compared with the punctures without the SIVA guide™ in the AL-horizontal (with 0 (0-1) times vs. 0 (0-0) time without the SIVA guide™; p=0.027) and AL-right 45-degree groups (with 0.5 (0-1) times vs. 0 (0-0) time without the SIVA guide™; p=0.013), but not in the AL-left 45-degree group (with 0 (0-1.25) times vs. 0 (0-0) time without the SIVA guide™; p=0.058) (Figure 4).

**Figure 5:**
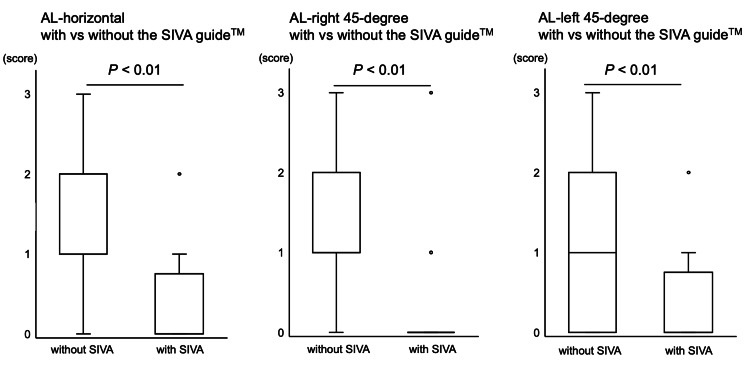
The needle image quality The needle image quality under the conditions with and without the SIVA guide™ in AL-horizontal, AL-left 45-degree, and AL-right 45-degree groups, respectively, is shown. The image evaluator classified the needle image quality as excellent (score 0), good (score 1), unsatisfactory (score 2), and poor (score 3). The data are shown as box-and-whisker plots (maximum and minimum values and IQR) with outliers (white circles).

The needle image quality score using a four-point score in the punctures with the SIVA guide™ was better compared with the punctures without the SIVA guide™ under all puncture settings (AL-horizontal with 2 (1-2) vs. 0 (0-0.25) without the SIVA guide™ (p<0.01); AL-right 45-degree with 1 (1-2) vs. 0 (0-0) without the SIVA guide™ (p<0.01); AL-left 45-degree with 1 (0-1) vs. 1 (0-0.25) without the SIVA guide™ (p<0.01)) (Figure 5).

**Figure 6:**
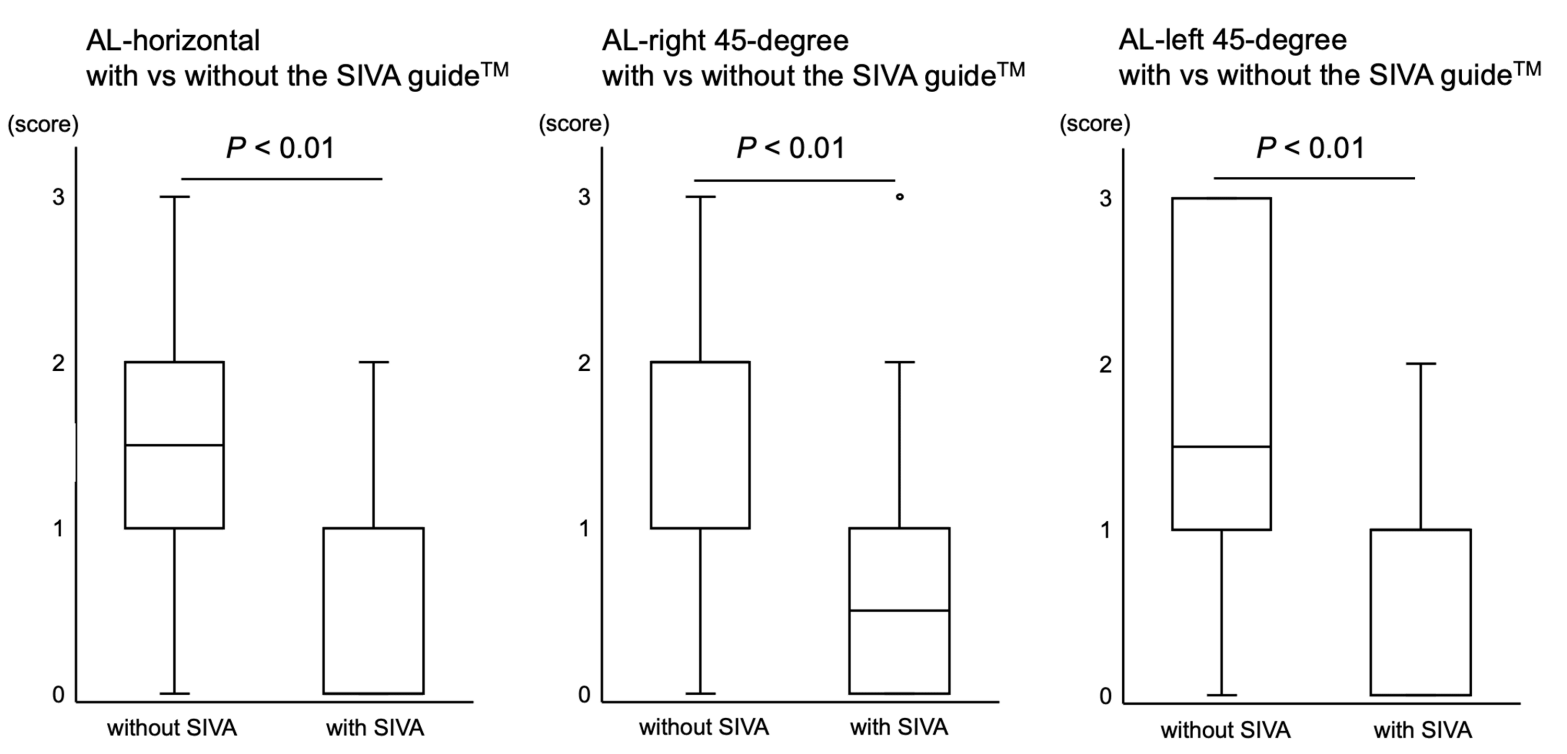
The procedure difficulty The participants' self-assessment of the procedure difficulty with and without the SIVA guide™ in AL-horizontal, AL-left 45-degree, and AL-right 45-degree groups, respectively, is shown. The participants evaluated the procedure difficulty as easy (score 0), normal (score 1), difficult (score 2), and extremely difficult (score 3). The data are shown as box-and-whisker plots (maximum and minimum values and IQR) with outliers (white circles).

The participants' self-assessment of the procedure difficulty score using a four-point score in the punctures with the SIVA guide™ was better compared with the punctures without the SIVA guide™ under all puncture settings (AL-horizontal with 1.5 (1-2) vs. 0 (0-1) without the SIVA guide™ (p<0.01); AL-right 45-degree with 2 (1-2) vs. 0.5 (0-1) without the SIVA guide™ (p<0.01); AL-left 45-degree with 1.5 (1-3) vs. 1 (0-1) without the SIVA guide™ (p<0.01)) (Figure 6).

## Discussion

We require minimal eye movement from the ultrasound monitor display when the display and the puncture direction are aligned straight during the ultrasound-guided in-plane puncture [6,11,12]. Nevertheless, obstacles such as a bed and a wall and the patient's position disturb the ideal puncture setting in clinical conditions. For example, when performing an internal jugular vein puncture using the parallel technique, the skin surface is not horizontal to the ground. However, all previous studies [8-10] about the usefulness of needle guidance devices in the in-plane ultrasound-guided puncture were implemented in conditions where the phantom surface was horizontally oriented. Therefore, we have conducted the present study employing novice medical students to show whether a needle guidance device improves the in-plane ultrasound-guided puncture quality in horizontal and inclined surface conditions to simulate clinical conditions. Indeed, our study firstly demonstrated that the time reaching the set success point of the target and the number of needle direction changes were less in the ultrasound-guided punctures with the SIVA guide™ compared with the punctures without the device in both horizontal and inclined surface conditions.

In the current study, the median times to reach the set success point between punctures with and without the SIVA guide™ were up to four-second time differences in all surface conditions, and the number of needle direction change differences in the punctures with and without the SIVA guide™ was small. On the other hand, the outliers in needle puncture speed were considerably larger without the SIVA guide™, suggesting that the needle guidance device use induces in-line puncture success uniformly, even in novice medical practitioners. Also, the needle image quality scores using a four-point score in the punctures with the SIVA guide™ were better than those without the SIVA guide™ under all puncture settings. These results agree with previous studies documenting that needle guidance devices facilitated the visualization and guidance of the needle in the ultrasound-guided in-plane approach using a horizontal surface [8-10]. Of these, two reports employed a commercially available device, Infinity™ (CIVCO Medical Solutions, Kalona, Iowa, United States) [8,9], and one used an original device [10]. Indeed, the previous results equivocally documented that needle guidance devices improved needle visualization on the ultrasound display [8-10]. We must note that novice medical practitioners performing ultrasound-guided nerve blocks exhibited poor ergonomics, constituting a critical mistake [7]. The most common error is the needle advancement without visualizing the needle tip, which happened in up to 44% of instances [7]. Simulator training has gained popularity in medical education [13], and the technology improves ultrasound-guided technical skills and knowledge acquisition [14]. However, two inappropriate techniques among novices, including the advancement of the needle before confirming visualization of the tip and the unintended probe movement, are still problematic in medical education [15]. Therefore, the needle guidance device may add some advantages to achieve a safer ultrasound-guided in-plane puncture, especially for novice practitioners. Indeed, the participants' self-assessment of the procedure difficulty score was better in the punctures with the SIVA guide™ compared with those without the needle guidance device under all puncture settings, indicating some satisfaction of novice practitioners in the current simulation study and the use of the needle guidance device for this purpose.

We have to mention several limitations in the current study. First, the present study employed a gel phantom as a simulation setting, and thus, the overall results may not reflect the ultrasound-guided in-plane needle puncture in human subjects. A gel phantom offers low impedance with a uniform background upon ultrasound use, and the needle adopted in the current study was somewhat echogenic [16]. Therefore, the above factors may bias the study results. Second, we evaluated the SIVA guide™ only as a needle guidance device in the current study. Thus, using other needle guidance devices may result in different outcomes for ultrasound-guided in-plane needle punctures. Third, in this study, a needle guidance device was attached to the probe, and the needle was inserted from the side of the probe. In actual clinical practice, the needle may be inserted from a site away from the probe to improve needle visibility, but this situation was not evaluated in this study. The ability to visualize the puncture needle with the free hand also needs to be trained. Fourth, whether the needle guidance device utilization accelerates the ultrasound-guided in-plane puncture technique acquisition remains uncertain in the present simulation study since each learning curve of the practitioner must vary [13,17,18]. Further studies using various needle guidance devices in human subjects are required to overcome the above points.

## Conclusions

The present study with the novice medical students revealed that the time it took to reach the set success point of the target in the ultrasound-guided punctures with the SIVA guide™ was less than in the punctures without the device in the conditions of the horizontal plane of the phantom, the plane inclined to the right at 45 degrees, and the plane inclined to the left at 45 degrees. The needle image quality score and the participants' self-assessment of the procedure difficulty score in the punctures with the needle guidance device were better than those without it. Therefore, a needle guidance device appears to improve the needle puncture speed and the quality of the needle visualization in the ultrasound-guided in-plane technique on both horizontal and inclined surfaces by novice medical students, compared with freehand.
